# Sample Sizes Based on Weibull Distribution and Normal Distribution for FRP Tensile Coupon Test

**DOI:** 10.3390/ma12010126

**Published:** 2019-01-02

**Authors:** Yongxin Yang, Weijie Li, Wenshui Tang, Biao Li, Dengfeng Zhang

**Affiliations:** 1 Central Research Institute of Building and Construction Co. Ltd, MCC, Beijing 100088, China; yangyongxin@tsinghua.org.cn (Y.Y.); libiao@cribc.com (B.L.); 2 School of Civil Engineering, Wuhan University, Wuhan 430072, China; whutangwenshui@whu.edu.cn (W.T.); DFzhang312@whu.edu.cn (D.Z.)

**Keywords:** sample size, Weibull distribution, normal distribution, fiber reinforced polymer (FRP), tensile coupon test

## Abstract

Current guidelines stipulate a sample size of five for a tensile coupon test of fiber reinforced polymer (FRP) composites based on the assumption of a normal distribution and a sample coefficient of variation (COV) of 0.058. Increasing studies have validated that a Weibull distribution is more appropriate in characterizing the tensile properties of FRP. However, few efforts have been devoted to sample size evaluation based on a Weibull distribution. It is not clear if the Weibull distribution will result in a more conservative sample size value. In addition, the COV of FRP’s properties can vary from 5% to 15% in practice. In this study, the sample size based on a two-parameter Weibull distribution is compared with that based on a normal distribution. It is revealed that the Weibull distribution results in almost the same sample size as the normal distribution, which means that the sample size based on a normal distribution is applicable. For coupons with COVs varying from 0.05 to 0.20, the sample sizes range from less than 10 to more than 60. The use of only five coupons will lead to a prediction error of material property between 6.2% and 24.8% for COVs varying from 0.05 to 0.20.

## 1. Introduction

Fiber reinforced polymer (FRP) composites have been increasingly used in the strengthening of engineering structures due to their advantages of high tensile strength, excellent corrosion resistance, light weight, and flexibility in shape [1,2,3,4,5,6,7]. Studies have validated that the application of FRP composites can improve the flexural capacity [8,9], stiffness [10], fatigue performance [11,12,13], and corrosion resistance [14] of structures. FRPs are also very attractive in shear strengthening and confinement [15,16]. In order to accurately evaluate the strengthening effects of FRP composites, it is necessary to first derive the valid data of FRP’s properties. In addition, for structures strengthened with prestressed FRP composites, the acquisition of valid data is especially important, since the prestressing load might account for a non-negligible part of FRP’s bearing capacity [11,17,18,19]. An inaccurate appraisal of FRP’s tensile strength will expose the structures to higher risk of premature failure. Available guidelines stipulate a minimum value of five for FRP tensile coupon test [20,21,22,23]. This stipulation uses a normal distribution for the characterization of FRP’s properties, and assumes a value of 0.058 as the sample coefficient of variation (COV) [21]. It is expected that with five coupons, there will be a 95% confidence that the relative error between the sample mean and the true mean value will be less than 5%.

Although a normal distribution is assumed in current guidelines for the characterization of FRP’s tensile properties, many studies have revealed that a Weibull distribution is more appropriate as the describing model, especially for the tensile strength [24,25,26,27]. Zureick et al. compared the normal, log-normal, and Weibull distribution based on more than 600 samples, and recommended the Weibull distribution for the characterization of FRP’s tensile strength and tensile modulus [24]. The same research team also studied the two-parameter and three-parameter Weibull distribution, and recommended the two-parameter model after taking into account the fitting goodness and the computational efficiency [28]. Gomes et al. conducted tensile tests on 1368 coupon samples [27]. It is confirmed that an overall good fit can be achieved by any of the normal, log-normal, or Weibull distribution. Despite this, the Weibull distribution provides the best prediction results in the tail region, and therefore is more appropriate as the modelling distribution. The inaccuracy of other models in the tail region prediction was also validated by Sanchez-Heres et al. [29]. Atadero compared the normal, lognormal, Weibull, and gamma distributions using more than 900 samples. The results showed that the Weibull distribution had a slight advantage in characterizing the tensile strength of FRP composites [25,30]. In addition to these experimental justifications, there are also some theoretical reasons for the use of Weibull distribution. The Weibull distribution is based on a weakest-link theory, which predicts that the failure of specimens is due to the weakest link (or the largest flaw) [31]. This agrees with the failure mechanism of FRP composites, and contributes to the use of Weibull distribution for property modelling. In comparison, the advantages of the normal distribution lie mostly in its ease of understanding and the availability of a closed-form analysis. It is symmetric and therefore is not suitable for the characterization of many engineering properties which show skewness to some extent. So far, the Weibull distribution has been adopted in the Composite Materials Handbook-MIL [32] and used by many researchers for the design strength (the 5th percentile value) analysis of FRP composites [24,27,33,34]. Nevertheless, limited efforts have been devoted to the sample size analysis using a normal distribution for mean value assessment. Bain proposed a method through which the sample size depends only on the accuracy and the percentile of the Weibull distribution [31]. Bain’s study facilitated future research. However, the correspondence between the percentile and the mean value are not within the scope of Bain’s study. In addition, only one-side confidence interval is presented by Bain, whereas an exact two-side confidence interval is not available. To the best knowledge of the authors, the sample size based on a Weibull distribution is still not available. It is not clear if the sample size provided by the Weibull distribution is more conservative than that by the normal distribution.

Besides the selection of the distribution type, the COV value assumed in current guidelines is also inappropriate. The available guidelines assume a maximum value of 0.058 as the sample COV. However, many studies show that the variation can be between 5% and 15% [33,35,36,37]. The sample size of five based on an assumption of 0.058 cannot ensure the accuracy of samples when the sample COV is higher than 0.058.

This study aims to present a sample size analysis based on the Weibull and normal distribution. A confidence level of 95% and a relative error limit of 5% were used for analysis, in accordance with stipulations in current guidelines. The sample sizes based on the Weibull and normal distributions were presented and the conservativeness of the two distributions was compared. The effects of COV on the sample size determination were also revealed. In addition, the accuracy of using only five coupons for COVs ranging from 0.5 to 2.0 were analyzed. It is expected the sample size analysis will facilitate researchers and engineers in choosing the sample size for FRP tensile test and provide reference for specification in FRP guidelines. In addition, the maximum COV discussed by the authors is as high as 0.2. This will be beneficial for experiments exposed to severe environments, where the COVs of FRP’s properties may be high [38]. 

## 2. Sample Size Based on Weibull Distribution

### 2.1. Introduction to Weibull Distribution

In order to facilitate further discussion, it is necessary to first present an introduction to the Weibull distribution. The probability density function (PDF) of a two-parameter Weibull distribution is:

(1) $$f(x)=\frac{\beta }{\theta }{\left(\frac{x}{\theta }\right)}^{\beta -1}\exp\left[-{\left(\frac{x}{\theta }\right)}^{\beta }\right]\;x\geq 0,\;\theta >0,\;\beta >0$$

where *θ* and *β* are termed as the scale and shape parameters, respectively. The corresponding cumulative distribution function (CDF) is:

(2) $$F(x)=1-\exp\left[-{\left(\frac{x}{\theta }\right)}^{\beta }\right]\;x\geq 0,\;\theta >0,\;\beta >0$$

The mean value, *μ*, and the coefficient of variation, COV, of the Weibull distribution are:

(3) μ=θ Γ(1+1/β)

(4) $$\mathrm{COV}=\frac{\sqrt{\Gamma (1+\frac{2}{\beta })-\Gamma^{2}(1+\frac{2}{\beta })}}{\Gamma (1+\frac{1}{\beta })}$$

where Γ(·) is the gamma function.

By virtue of Equation (2), we can have the *p*-percentile value *x*_p_, for which $P[x<x_{p}]=p=F(x_{p})$. The expression for *x*_p_ is:

(5) $$x_{p}=\theta {\left[-\ln\left(1-p\right)\right]}^{1/\beta }$$

### 2.2. Percentile of the Mean Value 

Bain proposed a method through which the sample size depends only on the p-percentile, the confidence level, and the desired confidence interval [31], as mentioned in the Introduction. Since we focus on the mean value, it is necessary to ascertain the percentile of the mean value. In engineering practice, we commonly use the arithmetic mean value $\bar{X}$ (estimated through the method of moments) as an approximation of the true mean value *μ*. However, if the properties conform to a Weibull distribution, a more exact estimation of *μ* is the maximum likelihood estimation (MLE) estimator $\hat{\mu }$, the value of which is derived based on the MLE estimation of *θ* and *β*, denoted as $\hat{\theta }$ and $\hat{\beta }$ [24]. The expression of MLE estimation for *θ* and *β* are as follows:

(6) $$\frac{\sum_{i=1}^{n}x_{i}^{\hat{\beta }}\ln(x_{i}^{})}{\sum_{i=1}^{n}x_{i}^{\hat{\beta }}}-\frac{1}{\hat{\beta }}-\frac{1}{n}\sum_{i=1}^{n}\ln(x_{i}^{})=0$$

(7) $$\hat{\theta }={\left(\sum_{i=1}^{n}x_{i}^{\hat{\beta }}/n\right)}^{1/\hat{\beta }}$$

where *x*_i_ are the sample values, *n* is the sample size, $\hat{\theta }$ and $\hat{\beta }$ are the estimators of *θ* and *β*. With $\hat{\theta }$ and $\hat{\beta }$, the MLE estimation $\hat{\mu }$ can be derived. The population distribution f(x), population COV, and *p*-percentile value *x*_p_ can also be inferenced, denoted as $\hat{f}(x)$, $C\hat{O}V$, and ${\hat{x}}_{p}$, respectively.

Let $\hat{\mu }$ be equal to ${\hat{x}}_{p}$. The percentile *p* of the estimated mean value $\hat{\mu }$, denoted as $p(\hat{\mu })$, of the distribution $\hat{f}(x)$ can be determined:

(8) $$p(\hat{\mu })=1-\exp\left\{-{\left[\Gamma \left(1+1/\hat{\beta }\right)\right]}^{\hat{\beta }}\right\}$$

It can be seen that the estimator $\hat{p}$ only depends on $\hat{\beta }$. In addition, it is noted from Equation (4) that the MLE estimate depends only on $\hat{\beta }$ as well. Therefore, the percentile $\hat{p}$ of the estimated mean value $\hat{\mu }$ is directly related to $C\hat{O}V$. In other words, once the $C\hat{O}V$ is derived, the percentile of the $\hat{\mu }$ can be determined. Based on Equations (4) and (8), the correspondence between the $C\hat{O}V$, $\hat{\beta }$, and the percentile *p* is presented in Table 1.

### 2.3. Sample Size Analysis

**Figure 1:**
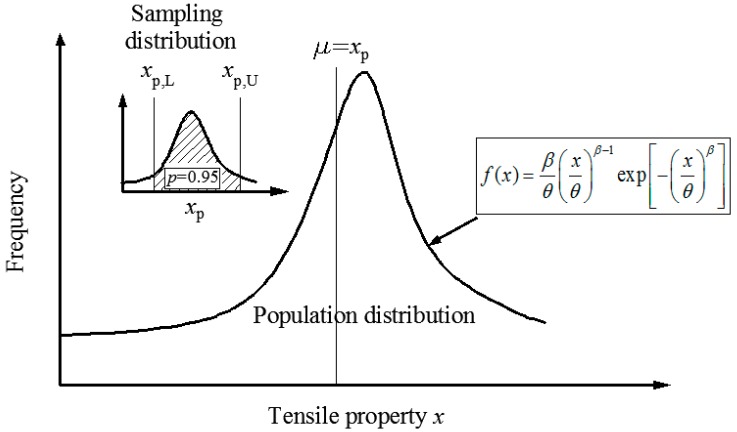
Illustration of the population distribution and the sampling distribution.

$\hat{\mu }$ is the inference on FRP’s property based on the results from limited sample sizes. In order to present $\hat{\mu }$ within a certain confidence interval, we need to have the distribution of $\hat{\mu }$ (or its corresponding percentile value ${\hat{x}}_{p}$). This is illustrated in Figure 1. Bain constructs a pivotal quantity to solve such a problem [31]. The pivotal quantity $U_{R}$ is defined as follows:

(9) $$U_{R}=\sqrt{n}\left[\ln\left(\frac{\ln R}{\ln\hat{R}}\right)\right]$$

where *n* is the sample size, R=1−p and is defined as the reliability, and $\hat{R}$ is the estimate of R, which depends on the estimated $\hat{\theta }$ and $\hat{\beta }$. The one-side confidence limit $U_{R,\;\gamma }$, for which $P[U_{R}<U_{R,\;\gamma }]=\gamma$, or two-side confidence limits, for which $P[U_{R,\;L}<U_{R}<U_{R,\;U}]=\gamma$, only depends on the confidence level γ, the percentile *p*, and the sample size *n*. Through Monte Carlo simulation, Bain presented the one-side confidence limit $U_{R,\;\gamma }$ for a series of γ and *p*, with the results listed in the Chapter 4, Table 4 of Bain [31]. The two-side percentage points are not available in Bain’s study. Therefore a Monte Carlo simulation was conducted by the authors to present the exact two-side confidence limits $U_{R,\;L}$ and $U_{R,\;U}$, using a commercial software, MATLAB (MathWorks, Natick, MA, USA). It was assumed that the confidence level was γ=0.95. The cases of $C\hat{O}V$ ranging from 0.05 to 0.20 were analyzed. The results of two-side confidence limits are shown Table 2. 

$U_{R}$ can also be expressed as a form related to $x_{p}$:

(10) $$U_{\gamma }=\sqrt{n}\hat{\beta }\ln\left({\hat{x}}_{p}/x_{p,\gamma }\right)$$

where $x_{p,\gamma }$ is the confidence limit of ${\hat{x}}_{p}$ for a given confidence level γ. For two-side confidence limit estimation, the lower and upper confidence limit are denoted as *x*_p, L_ and *x*_p, U_. By transforming Equation (10), the following formula can be derived:

(11) $$\frac{x_{p,\gamma }}{{\hat{x}}_{p}}=\exp\left(-\frac{U_{\gamma }}{\sqrt{n}\hat{\beta }}\right)$$

With Equation (11) and the two-side percentage points of $U_{\gamma }$ listed in Table 2, we can have the $x_{p,L}/{\hat{x}}_{p}$ and $x_{p,U}/{\hat{x}}_{p}$ for γ=0.95 and *p* equal to the values of $\hat{p}$ listed in Table 1, as presented in Table 3.

Our purpose is to ascertain the sample size, so that there is a γ confidence level that the inferenced mean value ${\hat{x}}_{p}$ will be within certain prediction error. The relative error between the estimated mean value ${\hat{x}}_{p}$ and the true mean value μ can be expressed as $\left|({\hat{x}}_{p}-\mu )/\mu \right|$. Since there is a γ confidence that ${\hat{x}}_{p}$ lies between $x_{p,L}$ and $x_{p,U}$, the relative error of ${\hat{x}}_{p}$ at γ confidence level is within $\max\left\{\left|x_{p,\;L}/\mu -1\right|,\;\left|x_{p,\;U}/\mu -1\right|\right\}$. As the true mean value *μ* of the population is not available, it is reasonable to substitute *μ* with its MLE estimation ${\hat{x}}_{p}$. Therefore, the relative error limit at a γ confidence level can be expressed as $\max\left\{\left|x_{p,\;L}/{\hat{x}}_{p}-1\right|,\;\left|x_{p,\;U}/{\hat{x}}_{p}-1\right|\right\}$.

By utilizing the data in Table 3, the sample size corresponding to various $C\hat{O}\mathrm{Vs}$ within a prediction error of 5% and with a confidence level of 95% can be ascertained, as shown in Table 4.

It is worth mentioning that, for engineering convenience, it is acceptable to use the sample COV (sample standard deviation divided by the sample mean) as the $C\hat{O}V$ in Table 4, which will facilitate the calculation on the sample size for the FRP coupon test [24]. 

## 3. Sample Size Based on Normal Distribution

Let *X*_1_, *X*_2_, …, and *X*_n_ be a sample taken from a normal distribution *N* (*μ*, *σ*^2^), where *n* is the sample size, and *μ* and *σ* are the mean and standard deviation of the normal distribution. The statistic $T=\frac{\left(\bar{X}-\mu \right)}{S/\sqrt{n}}$ follows a student’s t distribution with *n*−1 degrees of freedom, where $\bar{X}$ is the sample mean, and *S* is the sample standard deviation. The student’s t distribution is symmetric, and therefore the upper and lower confidence limit of T with a confidence level of γ (or 1−*α*, where *α* is termed as the significance level) are the 1−*α*/2 percentile value $t_{1-\alpha /2}(n-1)$ and *α*/2 percentile value $t_{\alpha /2}(n-1)$, respectively. Let $T=\frac{\left(\bar{X}-\mu \right)}{S/\sqrt{n}}$ be equal to $t_{1-\alpha /2}(n-1)$, the following equation can be derived:

(12) $$n={\left(\frac{t_{1-\frac{\alpha }{2}}(n-1)\times S}{\bar{X}-\mu }\right)}^{2}$$

This formula could be transformed as:

(13) $$n={\left(\frac{t_{1-\frac{\alpha }{2}}(n-1)\times \mathrm{COV}}{e}\right)}^{2}$$

where $\mathrm{COV}=S/\bar{X}$, representing the sample coefficient of variation. $e=\left|\bar{X}-\mu \right|/\bar{X}$ and denotes the relative error, or the accuracy. In fact, an exact equation for e is $e=\left|\bar{X}-\mu \right|/\mu$. However, as *μ* is not available, a practical method is to substitute *μ* with $\bar{X}$.

Equation (13) is an implicit equation for sample size *n*, due to the fact that the value $t_{1-\alpha /2}(n-1)$ also depends on *n*. *n* can be determined through trial and error method. Table 5 presents the sample size corresponding to varied COVs, with a confidence level of 0.95 and a relative error limit of 5%.

It is noted that all the values in Table 5 are higher than five, which is the value used in current guidelines. This result validates the risk in using only five coupons to derive the properties of FRP in tensile coupon test. In other words, the derived mean value based on five coupons might not meet the accuracy requirement of a relative error limit of 5%. It is especially interesting to note that even for a COV value of 0.05, the sample size, 7, is higher than the values used in current guidelines, 5. Theoretically, the sample size based on a COV of 0.05 should be less than the sample size stipulated in current guidelines, since the guidelines assume a slightly higher COV value, 0.058. A step-by-step explanation will be presented in Appendix A.

To reveal the prediction error if five coupons are used, Equation (13) is rearranged as the following:

(14) $$e=\frac{t_{1-\frac{\alpha }{2}}(n-1)\times \mathrm{COV}}{\sqrt{n}}$$

Based on Equation (14), the relative error limit of the derived mean value with various sample COVs can be illustrated (see Table 6):

## 4. Comparison and Recommendation

By comparing Table 4 with Table 5, it is found that the sample sizes based on the Weibull distribution and the normal distribution are almost the same, with the values based on the normal distribution being slightly larger. Strictly speaking, the sample sizes Table 4 cannot be directly compared with the values in Table 5, since the key parameter in Table 4 is the MLE estimator $C\hat{O}V$, whereas in Table 5 it is the sample standard deviation divided by the sample mean. However, since the $C\hat{O}V$ can be substituted by the sample COV for engineering convenience [24], such a comparison makes sense to some extent. The similarity in the sample sizes based on the Weibull distribution and the normal distribution shows that the Weibull distribution does not lead to a more conservative estimation on the sample size, and that sample size based on a normal distribution is applicable. 

For FRP coupon test, it is recommended that the sample sizes listed in Table 5 be used, i.e., 7 coupons for COV = 0.05, 18 coupons for COV = 0.10, etc. It is worth mentioning that if the coupons are with large COVs, the researchers should carefully check the fabrication and test procedure, rather than simply increasing the sample size to derive a more accurate property value. The large COVs can indicate problems with respect to the quality of fibers or resins, the impregnation procedure, the preparation and curation of specimens, the test setup, etc. Certain measures must be taken to correct the errors. The fabrication and test of samples should follow the procedure recommended by current guidelines [20,21,22,23].

## 5. Conclusions

This paper presents an analysis on the sample size for FRP coupon test. Both Weibull distribution and normal distribution were discussed with respect to the sample sizes corresponding to varied COVs. It was found that the sample size based on a Weibull distribution is almost the same as that based on a normal distribution (see Table 4 and Table 5). In other words, the Weibull distribution does not lead to a more conservative result with respect to the sample size for derived property with required accuracy and confidence level. Specifically, according to Table 4 and Table 5, the sample size is less than ten for a COV being 5% and more than 60 for a value after 20%. If only five specimens are used for tensile coupon test of FRP composites, the possible prediction error ranges from 6.2 to 24.8% when the COVs varies from 5% to 20%, which indicates that a minimum value of five cannot guarantee the accuracy for increased COVs.

## Figures and Tables

**Table 1:** Correspondence of $\hat{\beta }$, $\hat{p}$ and $C\hat{O}V$.

$C\hat{O}V$	0.05	0.1	0.15	0.2
$\hat{\beta }$	24.95	12.15	7.91	5.8
$\hat{p}$	0.4401	0.4507	0.4616	0.4728

**Table 2:** Two-side percentage points $U_{R,\;L}$ and $U_{R,\;U}$.

*n*	$\hat{p}$ $\;=44.01,\;C\hat{O}V\;=\;0.05$		$\hat{p}$ $\;=45.07,\;C\hat{O}V\;=\;0.10$		$\hat{p}$ $\;=46.16,C\hat{O}V\;=\;0.15$		$\hat{p}$ $\;=47.28,\;C\hat{O}V\;=\;0.20$	
	$U_{R,L}$	$U_{R,\;U}$	$U_{R,\;L}$	$U_{R,\;U}$	$U_{R,\;L}$	$U_{R,\;U}$	$U_{R,\;L}$	$U_{R,\;U}$
10	−2.5028	2.7384	−2.5154	2.7670	−2.4851	2.8421	−2.4602	2.9134
11	−2.4770	2.7047	−2.4885	2.7341	−2.4632	2.8040	−2.4459	2.8676
12	−2.4529	2.6732	−2.4634	2.7032	−2.4426	2.7683	−2.4322	2.8250
13	−2.4304	2.6436	−2.4401	2.6741	−2.4235	2.7349	−2.4192	2.7855
14	−2.4094	2.6160	−2.4184	2.6469	−2.4056	2.7038	−2.4067	2.7489
15	−2.3899	2.5902	−2.3983	2.6215	−2.3889	2.6747	−2.3949	2.7150
16	−2.3718	2.5661	−2.3798	2.5976	−2.3735	2.6477	−2.3836	2.6838
18	−2.3395	2.5229	−2.3469	2.5547	−2.3458	2.5993	−2.3626	2.6287
20	−2.3120	2.4857	−2.3192	2.5174	−2.3221	2.5578	−2.3437	2.5824
24	−2.2694	2.4268	−2.2771	2.4579	−2.2853	2.4927	−2.3116	2.5128
28	−2.2405	2.3851	−2.2495	2.4149	−2.2599	2.4474	−2.2861	2.4676
32	−2.2218	2.3565	−2.2327	2.3848	−2.2432	2.4170	−2.2664	2.4405
36	−2.2107	2.3375	−2.2235	2.3642	−2.2329	2.3976	−2.2514	2.4257
40	−2.2046	2.3251	−2.2192	2.3504	−2.2268	2.3856	−2.2405	2.4186
44	−2.2016	2.3167	−2.2176	2.3409	−2.2233	2.3781	−2.2328	2.4153
48	−2.1998	2.3103	−2.2169	2.3338	−2.2211	2.3727	−2.2277	2.4128
52	−2.1973	2.2993	−2.2139	2.3233	−2.2163	2.3641	−2.2227	2.4043
56	−2.1907	2.2920	−2.2082	2.3154	−2.2117	2.3553	−2.2201	2.3931
60	−2.1861	2.2835	−2.2053	2.3054	−2.2103	2.3438	−2.2189	2.3814
64	−2.1813	2.2768	−2.2001	2.2988	−2.2075	2.3351	−2.2186	2.3700
68	−2.1776	2.2727	−2.1942	2.2968	−2.2031	2.3314	−2.2194	2.3607

**Table 3:** $x_{p,L}/{\hat{x}}_{p}$ and $x_{p,U}/{\hat{x}}_{p}$.

*n*	$\hat{p}$ $\;=44.01,\;C\hat{O}V\;=\;0.05$		$\hat{p}$ $\;=45.07,\;C\hat{O}V\;=\;0.10$		$\hat{p}$ $\;=46.16,\;C\hat{O}V\;=\;0.15$		$\hat{p}$ $\;=47.28,\;C\hat{O}V\;=\;0.20$	
	$U_{R,\;L}$	$U_{R,\;U}$	$U_{R,\;L}$	$U_{R,\;U}$	$U_{R,\;L}$	$U_{R,\;U}$	$U_{R,\;L}$	$U_{R,\;U}$
10	0.966	1.032	0.931	1.068	0.893	1.104	0.853	1.144
11	0.968	1.030	0.934	1.064	0.899	1.098	0.862	1.136
12	0.970	1.029	0.938	1.060	0.904	1.093	0.869	1.129
13	0.971	1.027	0.941	1.057	0.909	1.089	0.875	1.123
14	0.972	1.026	0.943	1.055	0.913	1.085	0.881	1.117
15	0.974	1.025	0.946	1.052	0.916	1.081	0.886	1.113
16	0.975	1.024	0.948	1.050	0.920	1.078	0.891	1.108
18	0.976	1.022	0.952	1.047	0.925	1.072	0.899	1.101
20	0.978	1.021	0.955	1.044	0.930	1.068	0.905	1.095
24	0.980	1.019	0.960	1.039	0.938	1.061	0.915	1.085
28	0.982	1.017	0.963	1.036	0.943	1.055	0.923	1.077
32	0.983	1.016	0.966	1.033	0.947	1.051	0.928	1.072
36	0.985	1.015	0.968	1.031	0.951	1.048	0.933	1.067
40	0.985	1.014	0.970	1.029	0.953	1.046	0.936	1.063
44	0.986	1.013	0.971	1.028	0.956	1.043	0.939	1.060
48	0.987	1.013	0.973	1.027	0.958	1.041	0.942	1.057
52	0.987	1.012	0.974	1.026	0.959	1.040	0.944	1.055
56	0.988	1.012	0.975	1.025	0.961	1.038	0.946	1.052
60	0.988	1.011	0.976	1.024	0.962	1.037	0.948	1.051
64	0.989	1.011	0.977	1.023	0.964	1.035	0.950	1.049
68	0.989	1.011	0.977	1.022	0.965	1.034	0.952	1.047

**Table 4:** Sample size from the Weibull distribution.

$C\hat{O}V$	0.05	0.10	0.15	0.20
Sample size	<10	17	35	63

**Table 5:** Sample size from the normal distribution.

Sample COV	0.05	0.10	0.15	0.20
Sample size	7	18	37	64

**Table 6:** Relative error limit in using five coupons for tensile test.

Sample COV	0.05	0.10	0.15	0.20
Relative error limit	6.2%	12.4%	18.6%	24.8%

## Appendix A. Explanation for the Determination of Sample Size 5 and 7

Both the current guidelines and the authors assume a confidence level of 0.95 and a relative error limit of 5%.

The Commentary of Japanese guideline JSCE-E 531 presents an in-detail explanation for the derivation of sample size 5 for FRP tensile coupon test. The derivation by guidelines is based on a formula similar to but different from Equation (13), which is as follows:

(A1) $$n={\left(\frac{T\times \mathrm{COV}}{e}\right)}^{2}$$

*T*, which is 1.96 if the confidence level is 0.95, is used to substitute $t_{1-\frac{\alpha }{2}}(n-1)$ for the derivation of sample size by current guidelines. By using a COV value of 0.058, the sample size *n* is calculated as 5.2 based on Equation (A1). In the database of the guidelines’ collection, 0.058 is the maximum COV value for tensile strength, and the average value is 0.03. Therefore, the guidelines believe it is reasonable to use a sample size of 5 for tensile coupon test.

In comparison, the derivation by the authors strictly follows Equation (13). The sample size *n* is determined through trial and error method. For *n* = 7, the value $t_{1-\frac{\alpha }{2}}(n-1)$ = 2.4469, and the right-hand side of Equation (13) is 5.99. For *n* = 6, the value $t_{1-\frac{\alpha }{2}}(n-1)$ = 2.5706, and the right-hand side is 6.61. To ensure the precision and reliability of the mechanical property based on sample size *n*, the integer *n* should be higher than or equal to the value calculated in the right-hand side of Equation (13). Therefore, the sample size 7 is adopted by the authors for the case of COV = 0.05.
